# Host age affects the performance of the root hemiparasitic plant *Rhinanthus alectorolophus*

**DOI:** 10.1002/ece3.10167

**Published:** 2023-06-06

**Authors:** Belén Moncalvillo, Diethart Matthies

**Affiliations:** ^1^ Plant Ecology, Department of Biology Philipps‐Universität Marburg Marburg Germany

**Keywords:** host suppression, interactions, parasites, parasitic plants

## Abstract

Interactions between root hemiparasitic plants and their hosts are strongly affected by host identity, but may also depend on the condition of the host. An important determinant of host quality could be host age, as it may influence host size, allocation patterns, responses to infection, and the strength of competition for light between parasite and host. We investigated the effects of host species identity, host age and above‐ground separation of hemiparasite and host on the interactions between the hemiparasite *Rhinanthus alectorolophus* and five host species in a factorial experiment. The host species were planted at six different times, from 10 weeks before the parasite was planted to 4 weeks after. Host age strongly influenced the performance of the parasite, but these effects also varied among host species. Parasites grew largest with hosts planted at the same time or 2 weeks earlier, but their performance strongly declined both with increasing host age and with the time they grew autotrophically. A large part of the variation due to host age but not of that due to host species identity could be related to the negative influence of host size at the likely time of parasite attachment. The low quality of older hosts was not due to light competition, suggesting that effective exploitation of these hosts was prevented by other factors like harder roots, stronger defense against parasite attack or competition for resources taken up by the host roots. Suppression of host growth by the parasites declined with increasing host age. The results indicate that the choice of host age may influence the results of studies on hemiparasites. They also highlight the importance for annual root hemiparasites of attachment in early spring, that is, at a time when their mostly perennial hosts produce fresh roots but are still poorly developed above ground.

## Graphical abstract

Host age strongly influenced the performance of the hemiparasite Rhinanthus alectorolophus, and a large part of this variation could be related to the negative influence of host size at the likely time of parasite attachment. Suppression of host growth by the parasites decreased with increasing host age. The results indicate that the choice of host age may influence the results of studies on hemiparasites and highlight the importance for annual root hemiparasites of infection in early spring.

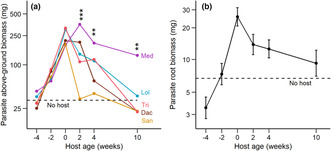

## 1 INTRODUCTION

About 4500 species of angiosperms belonging to at least 12 clades are parasitic (Těšitel, 2016). Parasitic plants extract water, nutrients, and carbon compounds from other plants by invading their shoots or roots with specialized organs called haustoria (Yoshida et al., 2016). While holoparasites are completely dependent on the resources provided by their hosts, hemiparasites are photosynthetically active (Westwood et al., 2010). Some root hemiparasites like *Rhinanthus* spp. are facultative parasites, that is, they can even grow and flower autotrophically without a host, but grow much larger with a host (Press, 1989). For the hosts, parasitism has often strong negative effects on their survival, biomass, and reproduction (Cameron et al., 2005; Press & Phoenix, 2005; Těšitel et al., 2010).

The identity of the host species can strongly influence the biomass, morphology, reproduction, and patterns of allometry of hemiparasitic plants (Campion‐Bourget, 1982; Jonstrup et al., 2016; Matthies, 2017, 2021; Press & Phoenix, 2005). There is also strong variation in the sensitivity of different host species to infection by hemiparasites (e.g., Cameron et al., 2009; Matthies, 2021). The interactions between root hemiparasites and their hosts are influenced by external factors, including nutrient and water availability (Korell et al., 2019; Mudrák & Lepš, 2010; Těšitel, Těšitelová, et al., 2015), levels of light (Matthies, 1995a; Těšitel et al., 2011), atmospheric CO_2_ concentration (Matthies & Egli, 1999), or mycorrhiza (Jung et al., 2012). However, interactions between parasitic plants and their hosts may also depend on the condition of the hosts, for example, its size at the time of infection (Matthies, 2017) or its damage by defoliation (Puustinen & Salonen, 1999).

A potential factor influencing hemiparasite–host interactions that has received little attention is the age of the host (Koch et al., 2004). A few studies that have investigated interactions between parasites and hosts of different ages used holoparasitic species and found that growth and reproduction of younger and smaller hosts were more strongly affected by parasitism than those of older hosts (Cechin & Press, 1993; Cirocco et al., 2020; Li et al., 2015; Seel & Press, 1996). The two studies focusing on parasite performance reported contrasting patterns. While the stem parasite *Cuscuta campestris* grew larger with older hosts (Koch et al., 2004), the root holoparasite *Striga hermonthica* produced more biomass with younger hosts (Gurney et al., 1999).

However, the effects of host age on parasite–host interactions can be expected to differ between holoparasites and hemiparasites, because hemiparasites rely to a large extent on their own photosynthesis for carbon uptake. Host plants are for hemiparasites sources of water and nutrients but also competitors for light (Matthies, 1995a; but see Matthies, 1995b). As older hosts are usually larger, they might be expected to be stronger competitors for light and thus influence the outcome of hemiparasite–host interactions. However, the single study on the effects of host age on root hemiparasite performance found that *R. minor* grew larger with 1‐year‐old than with 6‐week‐old individuals of the grass *Poa alpina* (Seel & Press, 1996), suggesting that a large source of resources was more important than the negative effect of shading. The single study on the effects of host age on host performance with a hemiparasite reported stronger negative effects of early infection (17‐ vs. 96‐day‐old hosts) by the root hemiparasite *R. minor* on the biomass produced by *Phleum bertolini* (Cameron et al., 2005). However, in that study the effects of age of the host at time of infection and of the duration of its growth with the parasite could not be separated.

**FIGURE 1:**
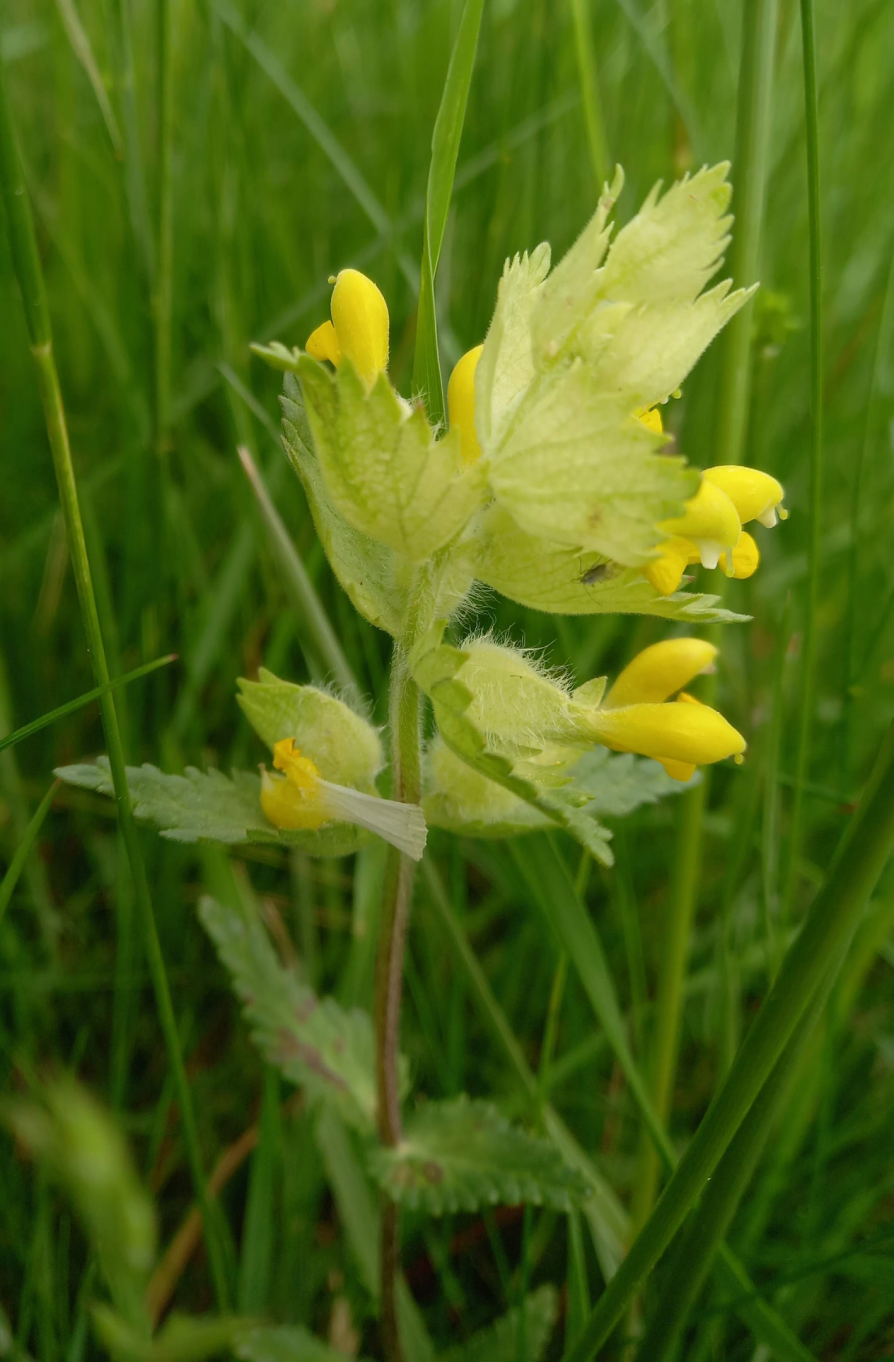
Photograph showing the study species *Rhinanthus alectorolophus*.

We grew the root hemiparasite *Rhinanthus alectorolophus* (Figure 1) with five different host species, each planted at six different times, from 10 weeks earlier to 4 weeks later than the parasite. These host treatments were combined with two treatments in which competition for light between hemiparasite and hosts was either allowed or prevented. To study early effects of host traits on parasite performance, we measured various host traits at the time of parasitic attachment for the different treatment combinations. Since *R. alectorolophus* is capable of autotrophic growth (Matthies & Egli, 1999), we also grew parasites without a host for comparison. To investigate the effect of the parasite on the hosts, hosts of different ages were also grown without a parasite. We asked the following questions: (1) What are the effects of host species identity and host age at the time of parasite planting on the performance of parasite and host? (2) How does competition for light affect the interactions between the hemiparasites and hosts of different ages? (3) Do host traits at the time of parasite attachment strongly influence final parasite performance?

## 2 MATERIALS AND METHODS

### 2.1 Study species

*Rhinanthus alectorolophus* (Scop.) Pollich (Orobanchaceae) is an annual facultative hemiparasite native to grasslands in Central Europe. It is a generalist which can use a wide range of species as hosts (Hautier et al., 2010; Matthies, 2021; Rowntree et al., 2011), and it is usually found in highly species diverse habitats, especially in low productivity areas with high light availability (Těšitel, 2016; Těšitel, Fibich, et al., 2015). *R. alectorolophus* was formerly considered a weed of cereal crops (Fürst, 1931), but nowadays is regarded as a keystone species or ecosystem engineer associated with the maintenance of biodiversity (Chaudron et al., 2021). Seeds of *R. alectorolophus* germinate in late autumn, but only the radicle develops during winter and cotyledons appear above ground in early spring. We selected five native perennial species as hosts which often co‐occur with *R. alectorolophus*: Two grasses (*Dactylis glomerata* and *Lolium perenne*; Poaceae), two legumes (*Medicago sativa* and *Trifolium pratense*; Fabaceae), and a non‐leguminous herb (*Sanguisorba minor*; Rosaceae), which will be referred to by their genus name in the following. Previous research has shown these species to be good hosts for *R. alectorolophus* (Matthies, 2021; Sandner & Matthies, 2018).

### 2.2 Experimental setup

Seeds of parasite and hosts were obtained from a commercial supplier (Appels Wilde Samen). To break the dormancy of *R. alectorolophus*, seeds were placed on moist filter paper in Petri dishes and kept for 3 months at 5°C until cotyledons had formed. Seeds of the hosts were germinated in Petri dishes at room temperature shortly before planting.

Pots of 11 × 11  × 12 cm were filled with a 2:1 mixture of commercial potting soil (TKS, Floragard, Oldenburg) and sand and provided with 100 mL of a 2 g L^−1^ solution of a commercial fertilizer (N:P:K = 14:7:14%; Hakaphos, Compo, Vienna). Because of limited space, two identical sets of pots were set up in two growth chambers. Plants were grown at a 20°C/15°C (day/night) temperature regime and 16 h of lighting. In chamber A, lighting was provided by LED plant lamps (Dual 360VR, Neusius), while in chamber B lighting was by 400 W sodium high pressure lamps (SonTAgro, Philips). Levels of photosynthetically active radiation were in both chambers c. 250 μmol^−1^ m^−2^ s^−1^ at the soil surface. Pots were watered regularly, and their position was randomized every 2 weeks within each chamber.

Two individuals of the same host species were planted in the center of each pot, at a distance of c. 3 cm from each other. Two individuals of *R. alectorolophus* were planted close together at an equal distance from the two host individuals. After 3 weeks, when parasite leaves had turned a darker green indicating attachment to the hosts (Klaren & Janssen, 1978) parasites were thinned to one per pot. Parasite seedlings that died during the first 2 weeks were replaced.

**FIGURE 2:**
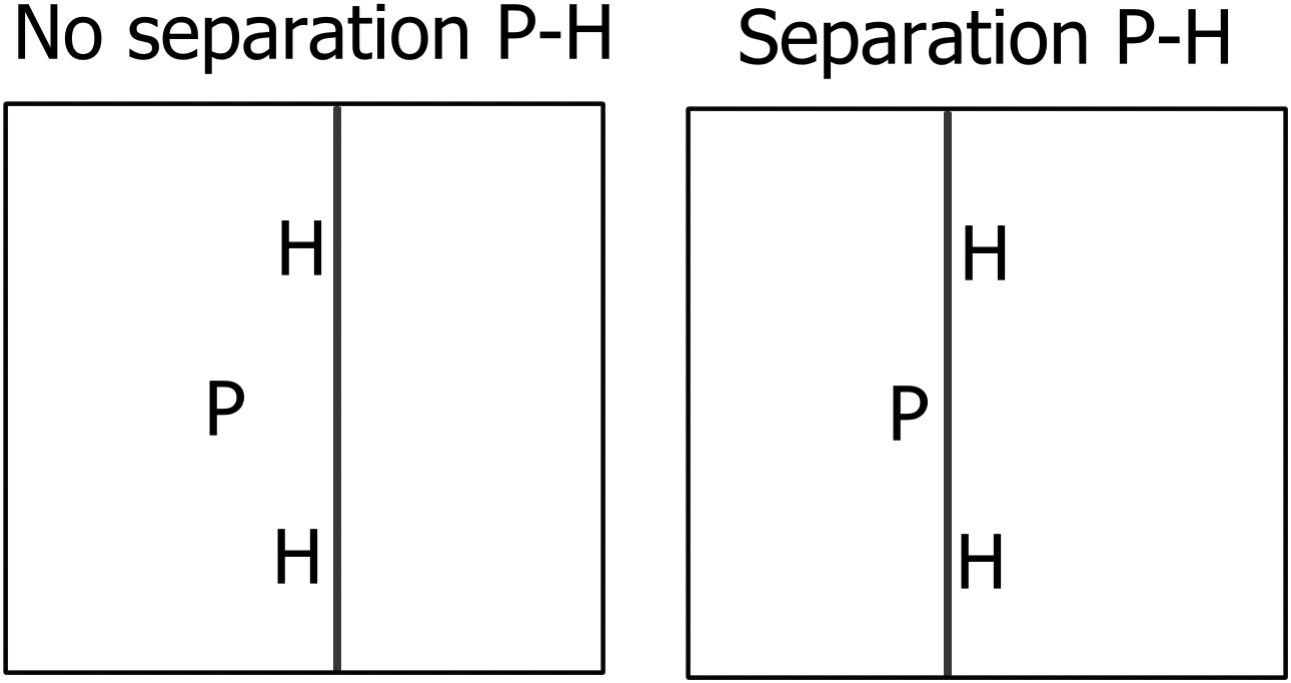
Schematic representation of the pots showing how parasites (P) and hosts (H) were either separated above ground by an aluminum foil (competition for light between hemiparasite and hosts not possible) or not (competition for light possible).

All parasites were planted on the same date, while their hosts were planted at six different times: 10 weeks earlier than the parasites (age 10 weeks), 4 weeks earlier (age 4 weeks), 2 weeks earlier (age 2 weeks), at the same time as the parasite (0 weeks), 2 weeks later (−2 weeks), and 4 weeks later (−4 weeks) than the parasite. Each pot was divided into halves above ground by a sheet of aluminum foil stretched between two stakes. In half of all pots, the division was placed between the hosts and the parasite to prevent competition for light between hemiparasite and hosts (Figure 2). In the other half of the pots, the division was placed in a way that hemiparasite and hosts were on the same side of the aluminum foil so that competition for light was possible. We set up 480 pots (5 host species × 6 host ages × 2 separation treatments × 2 chambers × 4 replicates).

To assess the effect of the parasites on the hosts, six pots per host species and age combination without a parasite were set up in addition (5 host species × 6 host ages × 2 chambers × 3 replicates = 180 pots) and to assess the autotrophic ability of *R. alectorolophus* 20 parasites were grown without a host (10 parasites per chamber). These two additional sets of pots were also divided by an aluminum foil into halves and the plants were grown on one side of it.

Early traits of the hosts may influence parasite performance and thus explain effects of host species identity and age. Therefore, two more pots for each combination of host species, chamber, and age 10, 4, 2, and 0 weeks were set up without a parasite to assess the size and other traits of the hosts at the time when the parasite started to attach to the hosts. This set included 5 host species × 4 host ages × 2 chambers × 2 replicates = 80 pots. In these pots the host plants were cut at ground level when the parasite had grown for 2 weeks in the other pots. The roots were carefully washed free of soil. All plant material was dried for 48 h at 80°C and weighed.

### 2.3 Measurements

To follow the development of parasites in the main experiment, the length of their longest leaf was recorded 3, 5, 7, and 9 weeks after planting. After 9 weeks of growth, when *R. alectorolophus* was at the peak of flowering, the following traits were measured for each parasite: height, total branch length (sum of the length of all branches plus the height of a plant), and the number of flowers. Parasites and hosts were then harvested separately above ground. For a subset of the pots, the roots of parasites and hosts were washed free of soil, dried and weighed. This subset consisted of four replicates of all combinations of host species and age (all with a parasite and separated; 120 pots) and of two replicates per combination of host species and age without a parasite (60 pots). In addition, the roots of four parasites grown without a host were also harvested. All plant material was dried for 48 h at 80°C and weighed.

### 2.4 Data analysis

The effects of the experimental treatments host age at the time of parasite planting, host species and above‐ground separation (to assess competition for light) of hemiparasite and host on (log) final parasite above‐ground biomass and the proportion of biomass allocated to roots (root mass fraction, RMF) was investigated by factorial analysis of variance. Chamber identity was included in the model as a fixed block factor. The relationships between parasite shoot mass and height, total branch length, number of flowers, length of the longest leaf, root mass, and RMF were analyzed by separate regressions.

We used a linear mixed model (R‐package lme4; Bates et al., 2015) to assess the effect of experimental treatments on the development of the length of the longest leaf of the parasites over time. In this model, parasite individual was included as a random factor to account for the repeated measurements of leaf length. To study the effect of host traits at a time when the parasites had grown for 2 weeks, we related mean final parasite shoot mass calculated per combination of host species, host age and chamber to mean total biomass of the hosts, mean above‐ground biomass and mean root mass with single regressions. We also investigated how much of the observed effects of host age and host species on final parasite biomass could be related to variation in these early host traits. For instance, for an analysis of the effect of early host mean total biomass, we carried out general linear models with sequential sums of squares of the effect of host age and species on mean final parasite mass. We then included mean early total biomass and its interaction with host age in this model, fitted them before the studied factors and calculated the proportional reduction in the variation due to the main effect of host species. To analyze the reduction in the variation in final parasite mass due to host age, we fitted the effects of mean early total biomass and its interaction with host species first. The effects of including the other early traits were analyzed analogously. The effects of parasite presence, host age and species on host biomass, host RMF, and total above‐ground productivity per pot were studied by factorial analysis of variance.

All analyses were carried out with *R* version 4.2.1 (R Core Team, 2022).

## 3 RESULTS

### 3.1 Parasite performance

**FIGURE 3:**
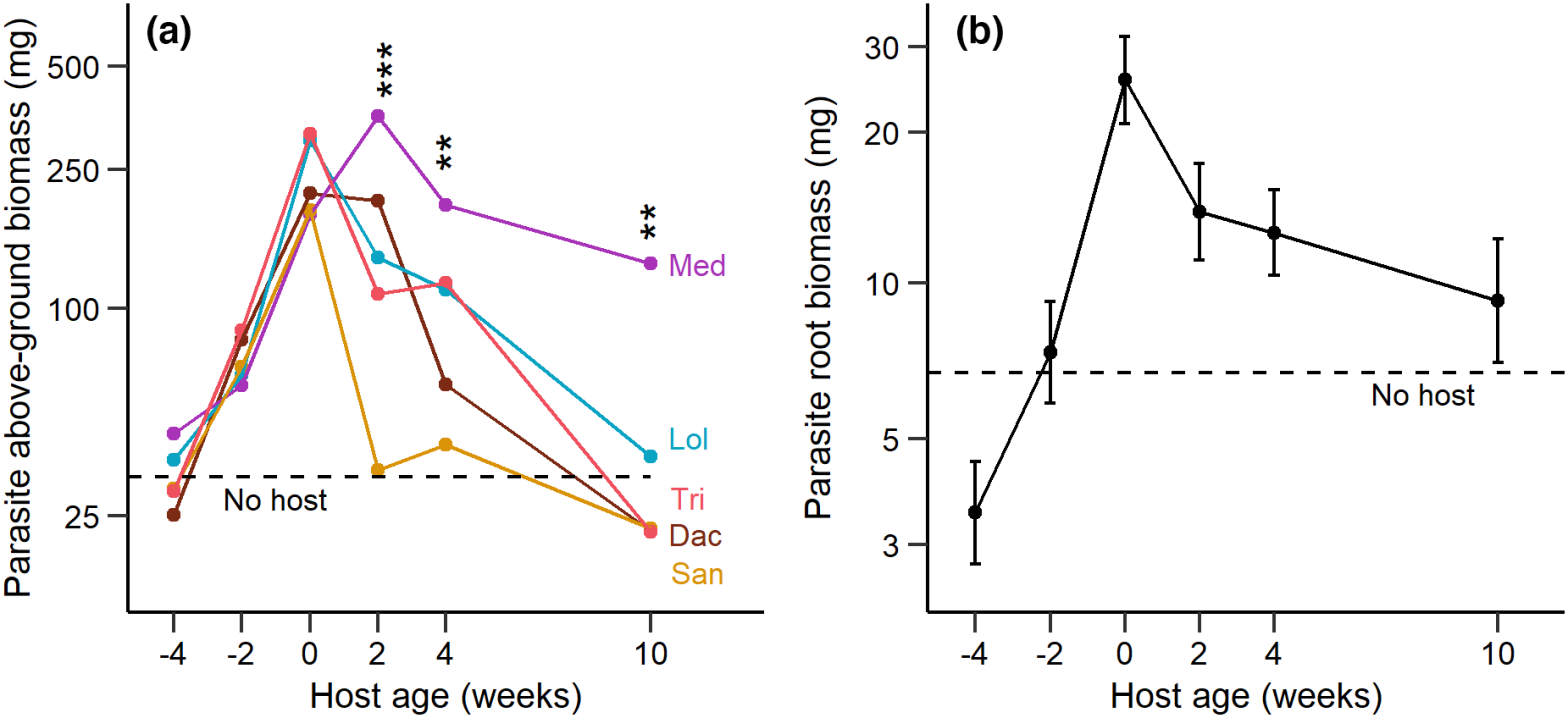
Effects of host species and host age at the time of parasite planting on (a) the above‐ground biomass, and (b) the root mass of the hemiparasite *Rhinanthus alectorolophus*. Significant differences among hosts at the same age are indicated as: **, *p* < .01; ***, *p* < .001. Dac, *Dactylis glomerata*; Lol, *Lolium perenne*; Med, *Medicago sativa*; San, *Sanguisorba minor*; Tri, *Trifolium pratense*. The biomass of parasites grown without a host is shown for comparison and is indicated by the broken line.

The above‐ground biomass of the parasite *Rhinanthus alectorolophus* depended strongly on both host species identity and the age of the host at the time of parasite planting (Table 1). Parasite biomass was 169% higher with the best host *Medicago* than with the worst host *Sanguisorba*. With all host species except *Medicago*, parasites grew largest with hosts planted at the same time, while their performance strongly declined with decreasing and increasing host age (Figure 3a). In contrast to its growth with the other host species, parasite biomass with *Medicago* was highest when the hosts were 2 weeks old and the decline in parasite performance with increasing host age was far less strong than with the other host species. Above‐ground separation between the hemiparasites and their host species did not significantly influence parasite performance (Table 1). The root mass of the parasites was influenced in a similar way by host age as was parasite shoot mass (Figure 3b), but there were no significant differences due to the different host species.

**TABLE 1:** Analyses of variance of the effects of host species, age of the hosts at the time of parasite planting, and above‐ground separation of parasite and hosts on the above‐ground biomass, root mass, and root mass fraction (RMF) of the parasite *Rhinanthus alectorolophus*.

Source of variation	df	Log above‐ground biomass		Log root mass		RMF	
		df_Res_ = 376		df_Res_ = 68		df_Res_ = 68	
		*F*	*p*	*F*	*p*	*F*	*p*
Chamber	1	0.17	.676	0.09	.761	1.95	.167
Host species	4	8.35	**<.001**	0.50	.736	3.92	**.006**
Host age	5	32.28	**<.001**	9.25	**<.001**	4.25	**.002**
Separation	1	0.13	.718				
Species × Age	20	2.37	**<.001**	0.84	.664	1.62	.074
Species × Separation	4	1.39	.236				
Age × Separation	5	0.86	.511				
Species × Age × Separation	20	1.18	.269				

*P*‐values < .05 are in bold face.

**FIGURE 4:**
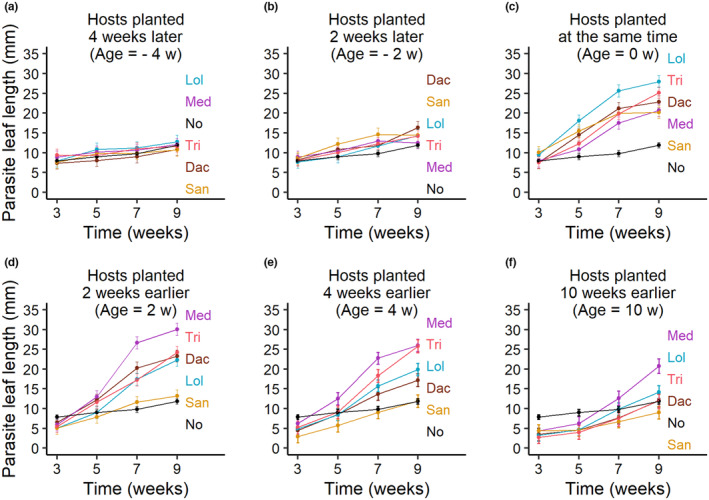
Development of the longest leaf (as a measure of plant size) of the parasite *Rhinanthus alectorolophus* over time when grown with different host species and hosts of different ages. (a) Hosts planted 4 weeks after the parasite, (b) hosts planted 2 weeks after the parasite, (c) hosts planted at the same time as the parasite, (d) hosts planted 2 weeks before the parasite, (e) hosts planted 4 weeks before the parasite, and (f) hosts planted 10 weeks before the parasite. In each figure, the development of parasites grown without a host is given for comparison. Means ±1 SE. The total number of measurements was *n* = 1822, plus 80 measurements for the parasites without a host. For abbreviations of host names, see Figure 3.

The length of the longest leaf of each parasite was followed over time to analyze differences in growth (Table 2, Figure 4). When the hosts were planted later than the parasites, there were no clear differences between the host treatments and this did not change over time (Figure 4a,b). However, when hosts and parasites were planted at the same time, differences among the parasites grown with different hosts developed already after 3 weeks of growth (Figure 4c). For parasites grown with older hosts, the effects of the different host species took longer to develop (Figure 4d–f).

**TABLE 2:** Mixed model analysis of the effects of the experimental treatments on parasite growth (estimated as length of the longest leaf) during the experiment.

Source of variation	df	*F*	*p*
Chamber	1	1.5	.219
Host age	5	32.2	**<.001**
Host species	4	6.4	**<.001**
Host age * Host species	20	3.1	**<.001**
Time	3	615.2	**<.001**
Time * Host age	15	30.5	**<.001**
Time * Host species	12	7.7	**<.001**
Time * Host age * Host species	60	2.2	**<.001**

*Note*: Parasite individual was included as a random effect in the model.

*P*‐values < .05 are in bold face.

**FIGURE 5:**
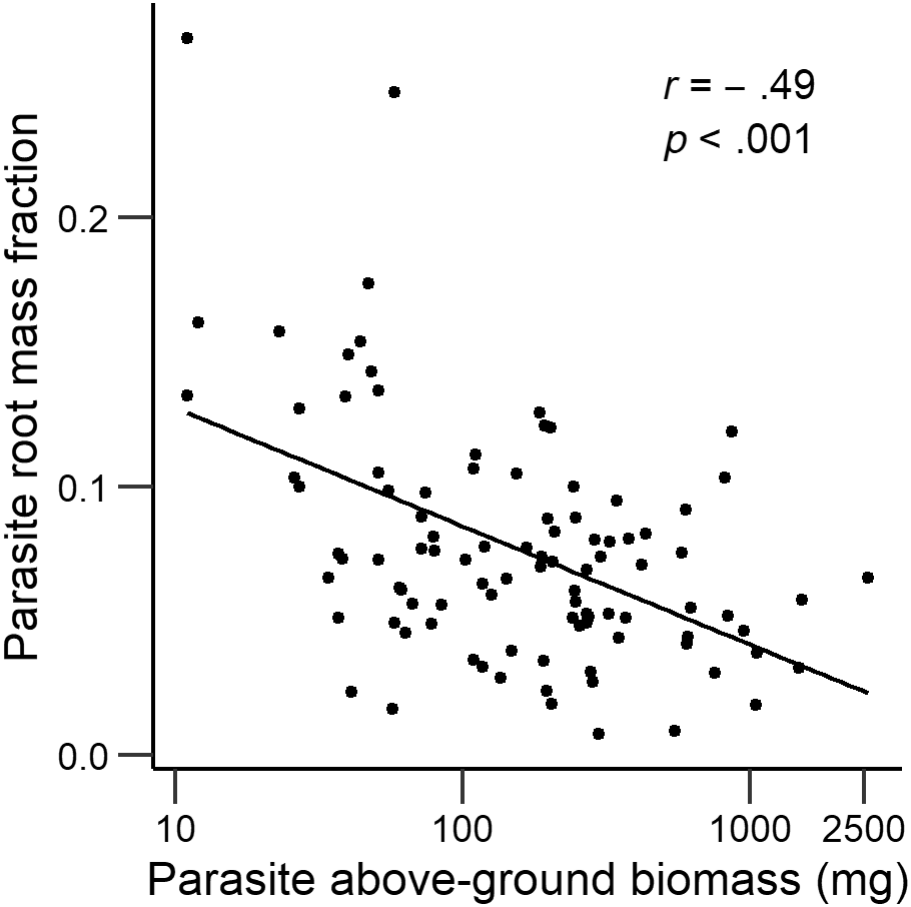
Relationship between the proportion of biomass allocated to roots (root mass fraction, RMF) by the parasite *Rhinanthus alectorolophus* and its above‐ground biomass.

**FIGURE 6:**
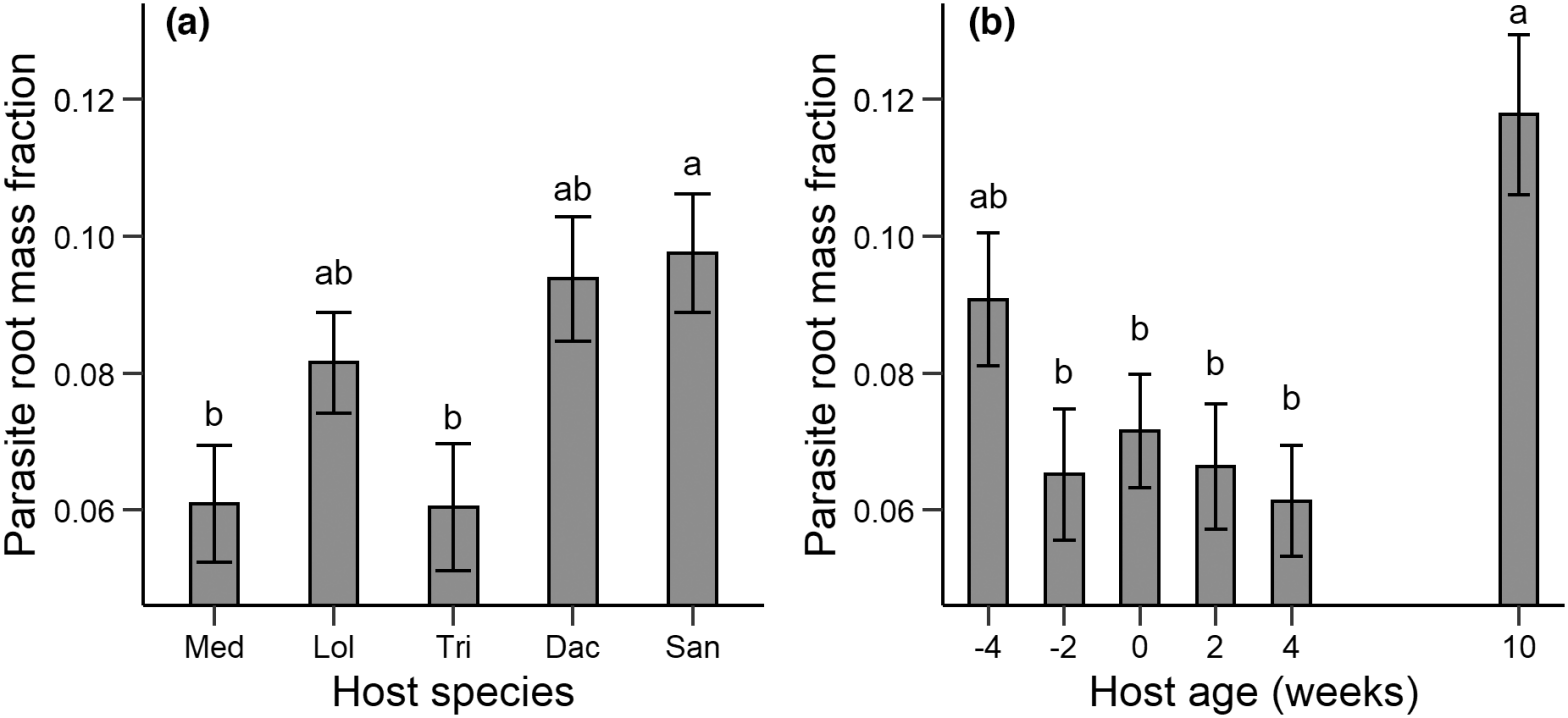
Effects of (a) host species and (b) host age at the time of parasite planting on the root mass fraction of the parasite *Rhinanthus alectorolophus*. For comparison, the mean RMF of parasites grown without a host was 0.17 ± 0.02. Host species are in order of decreasing final parasite above‐ground biomass. Means ±1 SE. Different letters indicate differences at the 0.05 level (Tukey test). For abbreviations of host names, see Figure 3.

Other parasite traits like height, total branch length, number of flowers, and leaf size were strongly positively correlated with above‐ground biomass (all *r* > .81, *p* < .001) and thus were similarly influenced by the experimental treatments. However, the larger the parasites were, the lower was the proportion of biomass they allocated to roots (Table 1, Figure 5). Consequently, parasite RMF was lowest with the two legumes *Medicago* and *Trifolium*, and highest with the worst host *Sanguisorba* (Figure 6a). Parasite RMF was lowest when grown with hosts that were planted at the same time as the parasite or a few weeks earlier or later (Figure 6b).

**FIGURE 7:**
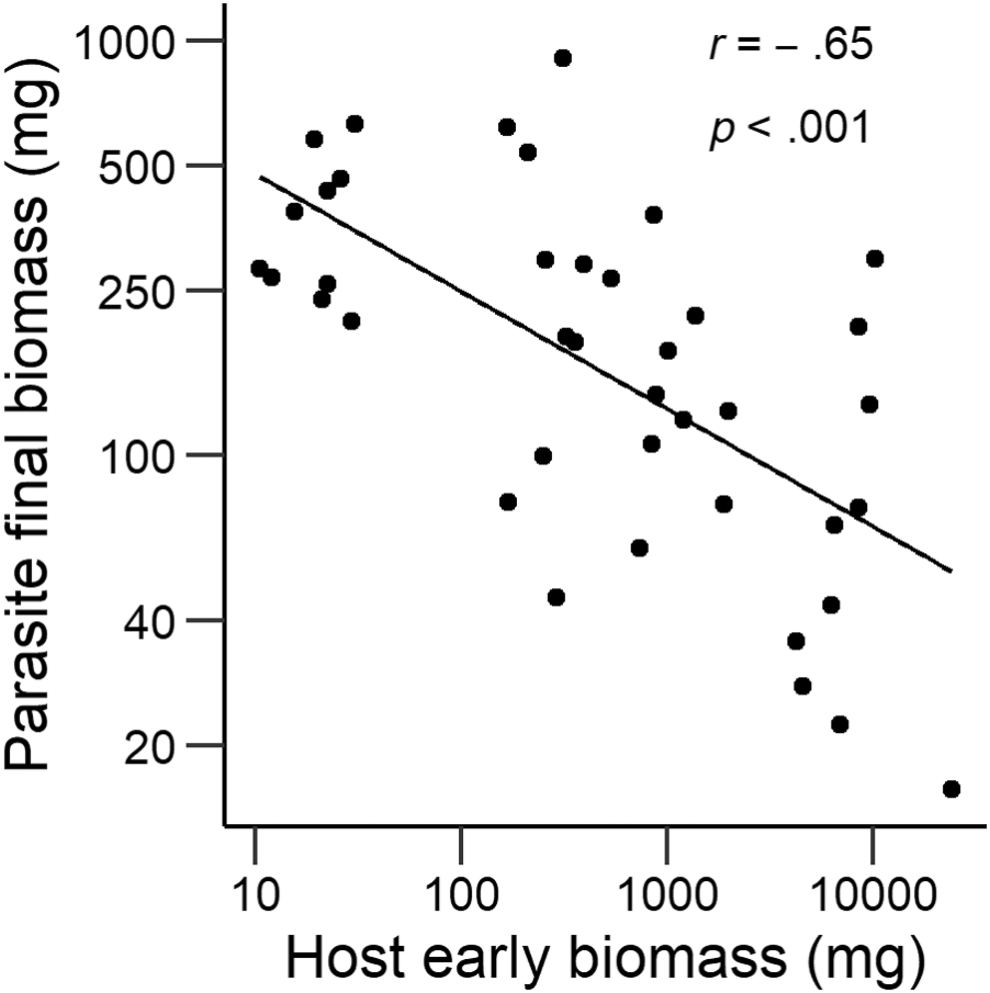
Relationship between the mean above‐ground biomass of the parasite *Rhinanthus alectorolophus* per treatment combination (host species × host age) in each chamber at the end of the experiment and mean total biomass of a separate set of hosts grown without a parasite that was harvested when the parasites in the other set had grown for 2 weeks.

Mean parasite above‐ground biomass at the end of the experiment was negatively related to several of the host mean trait values measured for a separate set of hosts 2 weeks after planting of the parasites. Mean above‐ground biomass of the hosts, root mass, and total host biomass each explained between 43% and 47% of the variation in final parasite mass (shown for total host mass in Figure 7). Variation in each of the mean host traits at Week 2 accounted for much (>93%) of the main effect of host age on mean final parasite biomass, but for much less of the main effect of host species identity (<16%). At the end of the experiment, the relationship between final parasite and final host biomass per pot was positive, but weak (*r* = .19, *p* < .001).

### 3.2 Effects of the parasite on host traits and total productivity

**FIGURE 8:**
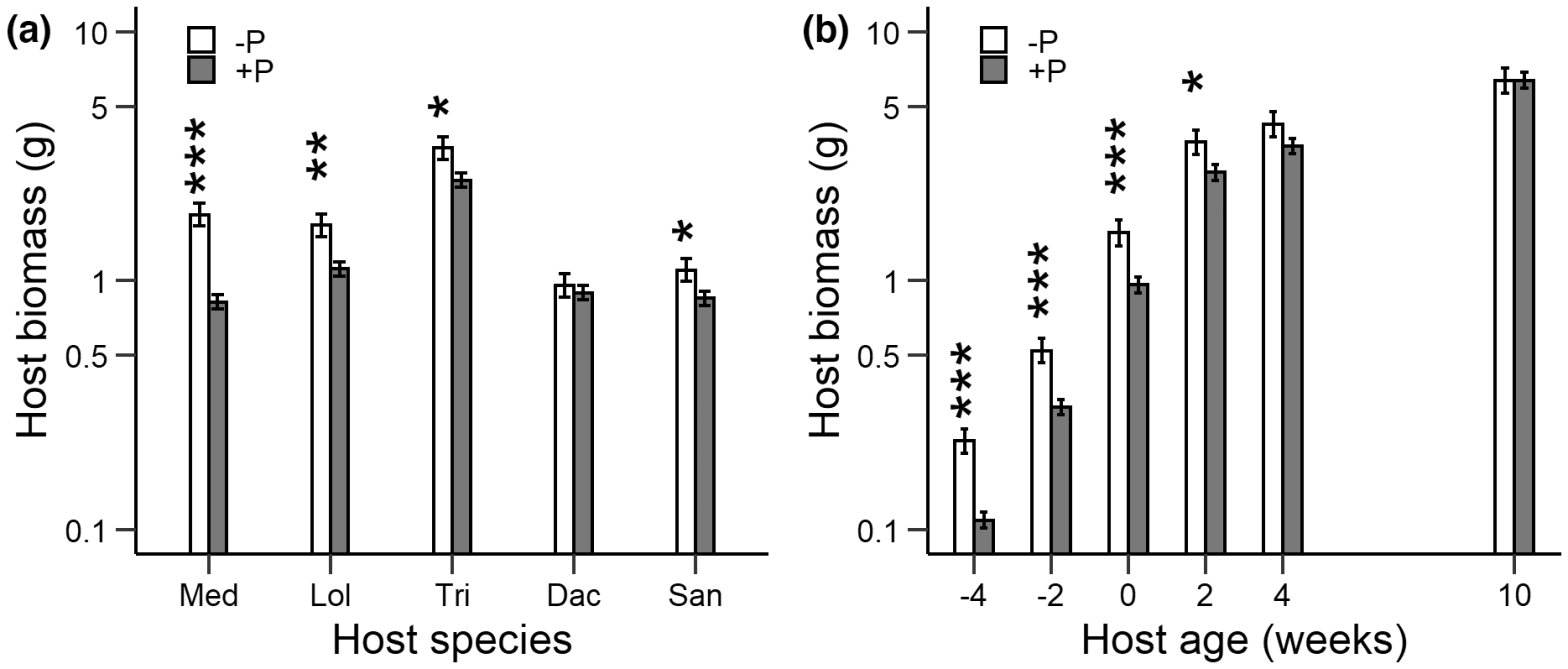
Above‐ground biomass of the hosts at the end of the experiment when grown with (+P) or without a parasite (−P). (a) The effect of the parasite on the five different host species; (b) the effect of the parasite on hosts of different age. Hosts had either already grown 10, 4, or 2 weeks without the parasites when the parasite was planted, hosts were planted at the same time as the parasite (age 0 weeks), or were planted 2 (age − 2) or 4 weeks (age − 4) after the parasite. *p*‐values for differences between the parasite treatments within (a) host species and (b) age treatments are indicated as: *, *p* < .05; **p < .01; ***p < .001.

The presence of the parasite *R. alectorolophus* reduced the above‐ground biomass of the hosts (Table 3), but the effect varied among host species from a 56% reduction for *Medicago* to only 6% for *Dactylis* (Figure 8a). The negative effect of the parasite on the hosts depended also on host age at the time of parasite planting (Figure 8b). Host suppression decreased with increasing host age from 52% for hosts planted 4 weeks later than the parasites to no reduction for hosts that were 10 weeks old when the parasites were planted. The parasites also affected the biomass allocation of the hosts. Hosts allocated a higher proportion of their biomass to roots when growing with a parasite (0.38 ± 0.01) than when growing alone (0.33 ± 0.01). There was no evidence that the effect of the parasite on host RMF was influenced by host species or age (all *p* > .46 for interactions with parasite presence; Table 3).

**TABLE 3:** Analyses of variance of the effects of host species, age of the hosts at the time of parasite planting, and the presence of the parasite *Rhinanthus alectorolophus* on above‐ground biomass and root mass fraction (RMF) of the hosts, and productivity per pot (above‐ground biomass of parasite + above‐ground biomass of host).

Source of variation	df	Log biomass		RMF		Productivity	
		df_Res_ = 595		df_Res_ = 110		df_Res_ = 594	
		*F*	*p*	*F*	*p*	*F*	*p*
Chamber	1	35.08	**<.001**	4.40	**.038**	30.58	**<.001**
Host species	4	56.73	**<.001**	11.72	**<.001**	65.71	**<.001**
Host age	5	444.40	**<.001**	13.36	**<.001**	492.19	**<.001**
Parasite presence	1	43.92	**<.001**	8.55	**.004**	11.27	**<.001**
Species × Age	20	7.93	**<.001**	0.87	.620	8.22	**<.001**
Species × Parasite	4	4.80	**<.001**	0.91	.462	3.35	**.010**
Age × Parasite	5	3.71	**.003**	0.85	.515	0.95	.450
Species × Age × Parasite	20	1.35	.143	0.68	.838	1.24	.215

*P*‐values < .05 are in bold face.

**FIGURE 9:**
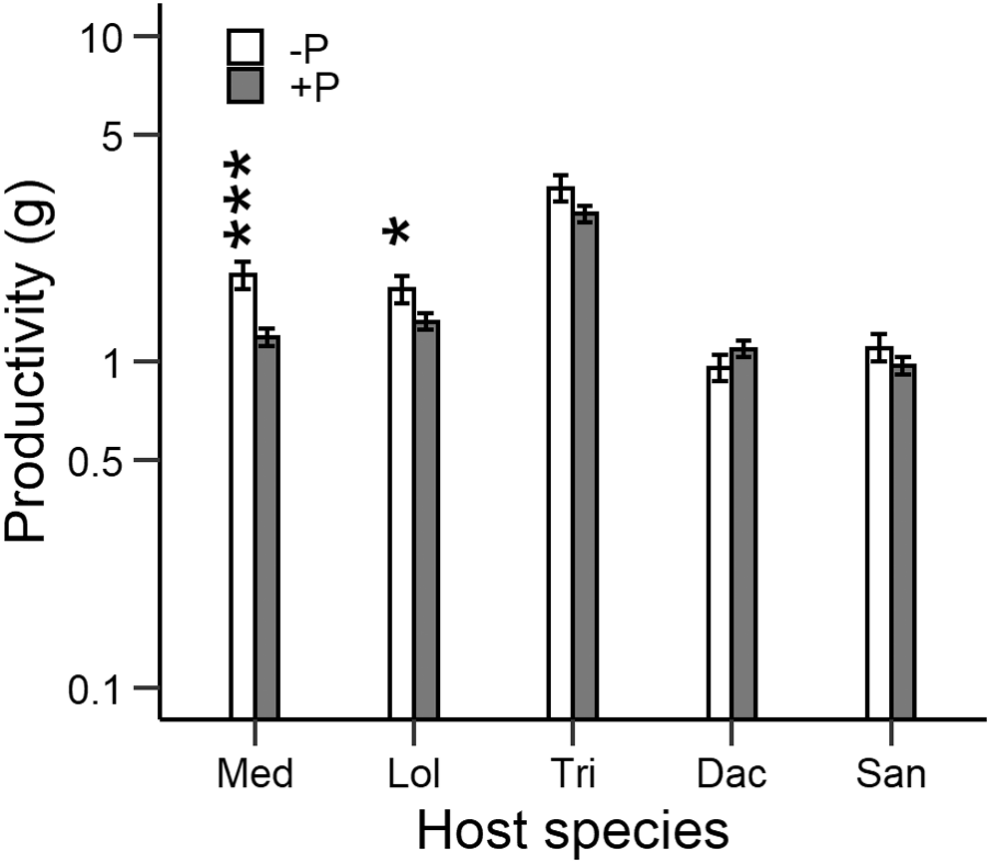
Productivity per pot (above‐ground biomass of parasite and hosts) at the end of the experiment when different host species were grown with the parasite *Rhinanthus alectorolophus* (+P) or without a parasite (−P). *p*‐values for differences between the parasite treatments within host species are indicated as: *, *p* < .05; *** *p* < .001.

Total productivity per pot (parasite plus host biomass) was affected by parasite presence (Table 3, Figure 9). Productivity was on average 15% lower in the pots with parasites, although this effect varied depending on species identity. Productivity was most strongly reduced with *Medicago* (−36%) and *Lolium* (−21%), whereas with the other host species there was no clear reduction in productivity (Figure 9).

## 4 DISCUSSION

Using a factorial experiment, we investigated the effects of host age and host species identity on the performance of the hemiparasite *Rhinanthus alectorolophus* and its hosts. Host age strongly influenced the performance of the parasite, but these effects varied among host species. A large proportion of the variation in final parasite biomass due to increasing host age, but not of that due to host species identity, could be explained by the negative influence of host size at the likely time of parasite attachment. The effects of the parasite on the hosts also varied depending on host age, as younger host plants were more strongly suppressed than older hosts.

### 4.1 Effects of host age on parasite performance

The performance of the hemiparasite *R. alectorolophus* was highest when grown with hosts that were planted at the same time as the parasite or 2 weeks earlier (except for *Sanguisorba*), but much poorer with hosts that were older (except for *Medicago*). Growth of the parasites was also poor with hosts that were planted 2 or 4 weeks later than the parasite, that is, were very young. In the case of the very young hosts, parasites had already grown for several weeks autotrophically and could have failed to attach to the newly planted hosts. However, the strong negative effect of the parasites on the growth of the young hosts shows that they had successfully infected the hosts. The small young hosts may not have provided sufficient nutrients, water and carbon for a strong growth of the parasites, as they represented a much smaller source than the older hosts (Cameron et al., 2005; Matthies, 2017). Moreover, these parasites only grew for 5–7 weeks with a host, whereas parasites from all other treatments had access to a host for 9 weeks. Benefits of an attachment to a host in the other treatments became only evident from after 5–6 weeks of growth of the parasite with a host.

In the other host age treatments all parasites grew for the same time with the hosts, but with hosts of different ages. Performance of those parasites more or less continuously decreased with increasing host age and was not higher than without a host with most of the 10‐week‐old hosts. This is in contrast to the positive effects of host age on parasite performance observed in *R. minor* grown either with 6‐week‐old *Poa alpina* or with hosts that had been planted in the year before (Seel & Press, 1996), in the holoparasite *Cuscuta campestris* grown with 3‐, 6‐ and 9‐week‐old *Trifolium resupinatum* (Koch et al., 2004), and in the holoparasite *Cuscuta australis* grown with 59‐, 74‐ and 83‐day‐old *Bidens pilosa* hosts. However, our results are in line with the higher performance of *Striga hermonthica* grown with young sorghum plants (18 days old) than with older host plants (28 days and 38 days old; Gurney et al., 1999).

There are several non‐mutually exclusive possible explanations for the general decline of parasite performance with increasing host age: (1) In pots with old hosts, nutrients may become depleted by the hosts, (2) older and thus larger hosts could suppress the growth of hemiparasites through strong competition for light, (3) roots of older host plants might be more difficult to penetrate by the parasites or show stronger defense reactions, and (4) shoots of older host plants could be very strong competitors for resources taken up by the host roots.

(1) A first possible explanation for the poor growth of the parasites with old hosts could be lack of nutrients, because the hosts might have taken up the available nutrients before the parasites were even planted. However, even 10‐week‐old hosts increased their mean biomass by more than 89% during the period of the parasite experiment, indicating that lack of nutrients in the soil was not the cause of the poor growth of parasites with old hosts.

(2) Root hemiparasites obtain most of their organic carbon from their own photosynthesis (Těšitel, Těšitelová, et al., 2015; Westwood et al., 2010), and they can therefore be sensitive to light competition, especially as seedlings (Těšitel et al., 2011). For example, competition for light by the host *Medicago sativa* reduced growth of the hemiparasites *R. serotinus* and *Odontites rubra* by more than 30% (Matthies, 1995a). The sensitivity of root hemiparasites to light competition is also shown by the fact that they are most abundant in habitats with open vegetation (Hejcman et al., 2011; Matthies, 1995a; Těšitel, Fibich, et al., 2015). However, in the present experiment there was no clear effect of above‐ground separation of hemiparasites and hosts on the performance of *R. alectorolophus*, indicating that competition for light was not the cause of the poor performance of the hemiparasites with the large older hosts (see also Matthies, 1995b). As differences in nutrient provisioning can have stronger impacts on parasite performance than above‐ground competition (Borowicz & Armstrong, 2012; Hwangbo & Seel, 2002), in the present study the effects of shading may have been insignificant in comparison with the large differences in the quantity of resources could extract from hosts of different ages. In addition, the shaded hemiparasites may have derived more carbon from their host and in this way may have compensated for their own reduced photosynthesis. Several studies have found that hemiparasites may obtain significant proportions of their carbon (up to more than 50%) from their hosts (Těšitel et al., 2010), in particular if they are shaded (Těšitel et al., 2011).

(3) A third explanation for the poor quality of older plants as hosts is a failure of the parasites to obtain sufficient resources from older hosts. This explanation is supported by the higher RMF of parasites grown with 10‐week‐old hosts. One of the main benefits of successful root parasitism is thought to be low investment of the parasites in their own root system (Fitter, 1986, review in Matthies, 2017). A high RMF suggests that parasites had either difficulties in successfully infecting host roots or in extracting solutes and thus invested more into their own roots (Press, 1989; Westwood, 2013). In our study, the more *R. alectorolophus* benefitted from a host, the less it invested in its own root system. Roots of the parasite have to encounter host roots, and parasite haustoria have then to penetrate several layers of host root tissue to establish contact with the host xylem (Kokla & Melnyk, 2018; Shen et al., 2006). If the root cortex of older plants is thicker and more lignified, it would make infection more difficult. The characteristics of host roots are known to influence haustoria formation (Riopel & Timko, 1995) and thicker host roots have been suggested as an explanation for differences in the performance of *R. minor* with the grass *Lolium perenne* grown under different conditions (Davies & Graves, 2000). Host plants can also actively defend themselves against the attack of parasitic plants, for example, by lignification, cell fragmentation, and the accumulation of toxic phenolic compounds (Cameron et al., 2006; Pérez‐De‐Luque et al., 2008). Defense reactions against plant parasites could increase with host age, in the same way as plants tend to invest more into constitutive defenses against herbivores later in life (Barton & Koricheva, 2010; Henn & Damschen, 2021). However, the species used as hosts in the current experiment were chosen because they had been shown to be good hosts for *R. alectorolophus* and to be little defended in previous studies (Matthies, 2021; Sandner & Matthies, 2018). They were also good hosts in the current experiment when they were attacked by the parasite at a certain age.

(4) Finally, it could be more difficult for the parasites to extract resources from older hosts that were already much larger than the parasites when the parasites started to extract solutes from the host roots. This explanation is supported by the observation that variation in the size of the host at the likely time of attachment explained a large proportion of the effect of host age on the final mass of *R. alectorolophus*. The flow of solutes to the parasite via the haustorium depends on a lower water potential in the parasite than in the host, which is achieved by particularly high transpiration rates of the parasites (Press et al., 1988; Shen et al., 2006; Yoshida et al., 2016). In large host plants with large canopies, competition between the parasite and the host shoot for resources taken up by the host roots might be severe as the parasites would need to counteract a lower water potential (Ehleringer & Marshall, 1995; Shen et al., 2006).

### 4.2 Effects of host species identity on parasite performance

The legume *Medicago* was a much more beneficial host for the parasite *R. alectorolophus* than the other species, in particular when the hosts were older than the parasite. Legumes such as *Medicago* spp. are usually very good hosts for hemiparasites (Matthies, 1996, 2017, 2021; Rowntree et al., 2014; Seel et al., 1993). The high quality of legumes as hosts has been attributed to their low resistance against infection by parasitic plants (Jiang et al., 2008; Rümer et al., 2007) and their high nitrogen content due to their association with nitrogen‐fixing rhizobia (Press et al., 1993). The acquisition of fixed nitrogen from the hosts has been considered to be the main advantage of parasitism for hemiparasites (Jiang et al., 2004; Westwood, 2013). The provision of a high quantity of nitrogen could also explain why *R. alectorolophus* allocated less mass to roots when grown with the legumes *Medicago* and *Trifolium* than with other hosts. However, *Trifolium* was a much poorer host than *Medicago*, indicating variation in host quality between members of the same functional group (Matthies, 2021; Rowntree et al., 2014).

In contrast to the effects of host age, the effects of host identity on parasite biomass could not be explained by host size at the likely time of parasite attachment. Thus, parasite performance with different hosts was more related to other likely differences among the species, such as strength of defense against parasitism (Cameron et al., 2006; Rümer et al., 2007) or the quality and quantity of solutes obtained by the parasite from them (Cameron & Seel, 2006; Jiang et al., 2004). In contrast, variation in the size of different host species at the time of parasite planting explained a considerable part of the variation in the final size of the related parasite *Melampyrum arvense* (Matthies, 2017), and initial host size had a positive effect on the growth of the stem parasite *Cassytha pubescens* (Cirocco et al., 2020).

### 4.3 Effects of the parasite on host biomass and total productivity

While *R. alectorolophus* strongly reduced the biomass of younger hosts, its relative effect decreased with increasing host age. Stronger effects on young hosts have also been reported for the root hemiparasite *R. minor* (Cameron et al., 2005; Seel & Press, 1996), the root holoparasite *Striga hermonthica* (Cechin & Press, 1993; Gurney et al., 1999), and the stem parasites *Cuscuta* spp. (Koch et al., 2004; Li et al., 2015). Similarly, small individuals of *Ulex europaeus* were more strongly affected by the stem parasite *Cassytha pubescens* than larger ones (Cirocco et al., 2020). *R. alectorolophus* may suppress younger hosts more strongly because it affects a larger part of the host root system and extracts a larger proportion of host resources than with older hosts (Cameron et al., 2005).

Host suppression by the parasite varied also strongly among species. While the biomass of *Dactylis* was hardly affected at all, that of the other hosts was reduced considerably, in particular that of *Medicago*. As both *Dactylis* and *Medicago* were good hosts for *R. alectorolophus*, this indicates that *Dactylis* was more tolerant of parasitism than *Medicago*. Moreover, in contrast to the results of some other studies (Li et al., 2012; Matthies, 1996, 2017; Sandner & Matthies, 2018) biomass of the hemiparasite with a host species and suppression of that host across age treatments were not positively related.

The parasite *R. alectorolophus* also influenced the root mass fraction of the hosts. Host plants allocated a greater proportion of their biomass to roots when growing with a parasite. This pattern has been observed in other studies (see Korell et al., 2019; and review in Matthies, 2017; but see Li et al., 2012) and can be interpreted as a mechanism to compensate for the loss of water and nutrients to the parasite (Matthies, 1995a) in line with the functional equilibrium hypothesis of biomass allocation in plants (Chapin, 1980). Alternatively, it could indicate active modification of host root morphology by the parasite through the transport of hormones from the parasite to the host roots via the haustoria (Spallek et al., 2017).

Negative effects of the parasite on total productivity per pot (host + parasite) were less general than those on host biomass and allocation. With some host species (*Sanguisorba*, *Trifolium*) the lower productivity of the hosts due to parasitism was compensated by the biomass produced by the parasite. In contrast, Hautier et al. (2010) predicted that total productivity would always be lower than that of the host growing without a parasite. While many studies have found a reduction of productivity by hemiparasites (Davies et al., 1997; Matthies, 1995a, 1995b; Phoenix & Press, 2005), this effect is not universal and, as in the current study, may depend on host species identity (Joshi et al., 2000; Matthies, 2017, 2021; Sandner & Matthies, 2018) or on nutrient availability (Haynes, 2021; Matthies & Egli, 1999).

## 5 CONCLUSIONS

The results of our study indicate that hemiparasites grow much poorer with hosts that are older than 2 weeks, and that host age influences the rank order of species in terms of their quality as hosts and the extent of suppression of the hosts. The choice of host age may thus affect the results of experimental studies on hemiparasite–host interactions. Most experiments have used hosts that were planted at the same time as the parasites (e.g., Irving et al., 2019; Matthies, 2017; Rowntree et al., 2011), 1–2 weeks later, or used hosts that were 1–2 weeks older than the parasites (Borowicz & Armstrong, 2012; Matthies, 2021; Ren et al., 2010; Sandner & Matthies, 2018; Těšitel et al., 2010). Our results suggest that growth of the hemiparasites with such hosts is strongest. However, hosts have also been sown several weeks before parasites were planted (Cameron et al., 2006) or planted in autumn and parasites planted in spring (Guo & Luo, 2010; Hautier et al., 2010; Jonstrup et al., 2016), thus more closely simulating the situation in the field.

In view of the poor growth of *R. alectorolophus* with older hosts it may seem surprising that many root hemiparasites grow in perennial communities where host plants are often many years old (Těšitel, Fibich, et al., 2015). However, annual hemiparasites like *Rhinanthus* spp., *Melampyrum* spp., *Euphrasia* spp., and *Odontites* spp. germinate and produce roots already in autumn or winter. The parasites thus may establish contact with freshly produced host roots in early spring at a time when host shoots and leaves are still poorly developed, produce leaves, and grow rapidly. It has been suggested (Fitter, 1986) that, since the hosts roots tend to die off in winter, the life history of annual root hemiparasites like *R. alectorolophus* may actually be a response to a seasonally available resource (roots).

## AUTHOR CONTRIBUTIONS

**Belén Moncalvillo:** Conceptualization (equal); data curation (equal); formal analysis (equal); writing – original draft (equal); writing – review and editing (equal). **Diethart Matthies:** Conceptualization (equal); data curation (equal); formal analysis (equal); writing – original draft (equal); writing – review and editing (equal).

## CONFLICT OF INTEREST STATEMENT

None declared.

## Data Availability

Data are available in the Dryad repository (https://doi.org/10.5061/dryad.c866t1gc0).
